# An Engineered Split Intein for Photoactivated Protein *Trans*-Splicing

**DOI:** 10.1371/journal.pone.0135965

**Published:** 2015-08-28

**Authors:** Stanley Wong, Abdullah A. Mosabbir, Kevin Truong

**Affiliations:** 1 Institute of Biomaterials and Biomedical Engineering, University of Toronto, 164 College Street, Toronto, Ontario, M5S 3G9, Canada; 2 Edward S. Rogers Sr. Department of Electrical and Computer Engineering, University of Toronto, 10 King’s College Circle, Toronto, Ontario, M5S 3G4, Canada; Imperial College London, UNITED KINGDOM

## Abstract

Protein splicing is mediated by inteins that auto-catalytically join two separated protein fragments with a peptide bond. Here we engineered a genetically encoded synthetic photoactivatable intein (named LOVInC), by using the light-sensitive LOV2 domain from *Avena sativa* as a switch to modulate the splicing activity of the split DnaE intein from *Nostoc punctiforme*. Periodic blue light illumination of LOVInC induced protein splicing activity in mammalian cells. To demonstrate the broad applicability of LOVInC, synthetic protein systems were engineered for the light-induced reassembly of several target proteins such as fluorescent protein markers, a dominant positive mutant of RhoA, caspase-7, and the genetically encoded Ca^2+^ indicator GCaMP2. Spatial precision of LOVInC was demonstrated by targeting activity to specific mammalian cells. Thus, LOVInC can serve as a general platform for engineering light-based control for modulating the activity of many different proteins.

## Introduction

Protein splicing is a unique post-translational phenomenon where an internal peptide sequence known as the intein, removes itself from a non-functional precursor protein while concurrently ligating the flanking precursor ends (i.e. exteins) with a peptide bond to restore the reassembled precursor gene’s function [1–3]. In exploiting this phenomenon, the ability to control protein splicing can offer new approaches of regulating protein activity. Currently there are few established evidence for naturally occurring regulators of protein splicing, which include recent research illustrating a redox-dependant mechanism of control [4,5]. Therefore, there are only a handful of natural clues to help guide potential engineered approaches. Protein splicing occurs in two modes: the more commonly found *cis* splicing mode where the intein is intramolecularly spliced from the host gene and the rarer *trans*-splicing mode where the inteins are naturally ‘split’ into two fragments that intermolecularly bind and then splice [6,7]. Through a combination of protein and chemical engineering strategies, artificially split *cis*-splicing inteins, which have significantly reduced affinity, have been regulated by temperature [8,9], pH [10], or small molecules [11–13]. However, temperature- and pH-sensitive inteins are limited to biological systems that can tolerate these changes while small molecules often mimic ‘drug-like’ compounds that can cause adverse side effects.

In contrast, light offers a potentially more desirable stimulus because at moderate dosages it causes minimal biological damage and it can be delivered with both temporal and spatial precision. Recently, other groups have developed systems of split inteins by using photocaged functional groups to the backbone [14,15] or side chain [16] to control protein synthesis and gene regulation [17–20]. Protecting groups were inserted into the protein backbone of split inteins to disrupt function by prohibiting intein binding. Subsequent UV stimulation dislodged the protecting group to restore intein binding. However, chemically caging the intein fragments require the use of protein synthesis and *ex vivo* modification to construct the precursors [14,15] or require the use of unnatural amino acids [16,20]. An alternative to chemical photocaging is genetically encoded light sensitive proteins. Genetically encoded photo-control of inteins has been previously demonstrated [21]. However, they used an artificially split intein that required an exogenous interacting domain to bring the two intein fragments together where the interacting domains were phytochrome proteins (PhyB and PIF3) which required the addition of tetrapyrrole co-factors during growth. In contrast, this study utilizes the light sensitive LOV (Light, Oxygen or Voltage) protein domain that does not require the exogenous addition of co-factors to control the spontaneous association of naturally split inteins. In particular, the LOV2 domain from *Avena sativa* phototropin has been shown to undergo large conformational changes when photo-stimulated with blue light [22–24]. In the dark state, the flavin-binding LOV2 domain associates with its carboxyl-terminal helical extension (Jα) in a tightly docked conformation [23,25]. Photoactivation of LOV2 leads to the formation of a covalent adduct between the flavin chromophore and the conserved Cys450 residue. This triggers a large conformational change that unwinds and undocks the Jα helix [23]. This conformational change has been used to engineer a photo-inducible synthetic bio-system in diverse target proteins such as DNA-binding proteins [26], enzymes [27], small GTPases [28], plasma membrane Ca^2+^ channels [29], and cell death [30].

Here, we engineered the LOV2 domain with the naturally split DnaE intein from *Nostoc punctiforme* (*Npu*DnaE) [31,32] to generate the genetically encoded photoactivatable protein *trans*-splicing (PTS) intein, named LOVInC. LOVInC is based on previous truncation design strategies [26,28–30] where the LOV2 effector domain acts as an allosteric switch to modulate activity of truncated target proteins. Thus, the LOV2 domain was fused to a truncated variant of the C-terminal *Npu*DnaE intein (InC) as a control mechanism to trigger PTS activity. In theory, the LOV2 domain in its dark-state closed conformation may allosterically interfere with InC and inhibit the spontaneous dimerization of the two *Npu*DnaE precursor fragments and block PTS activity. Upon illumination (lit-state), the Jα helix would release the inhibition and allow re-association of the intein precursors. As a demonstration, photoactivatable LOVInC was used to restore function to several target proteins: first, the light-induced reassembly of yellow fluorescent protein (i.e. Venus mutant [33]) to restore fluorescence; second, reassembly of the dominant positive mutant of RhoA GTPase (Q63L) [34] to induce dynamic blebbing in epithelial-like cells (i.e. HEK293 and HeLa); third, reassembly of caspase-7 to induce cell death morphologies; lastly, reassembly of the genetically encoded Ca^2+^ indicator GCaMP2 [35,36] to allow Ca^2+^ imaging.

## Results and Discussion

### Determining minimal functional C-terminal NpuDnaE fragment

**Fig 1:**
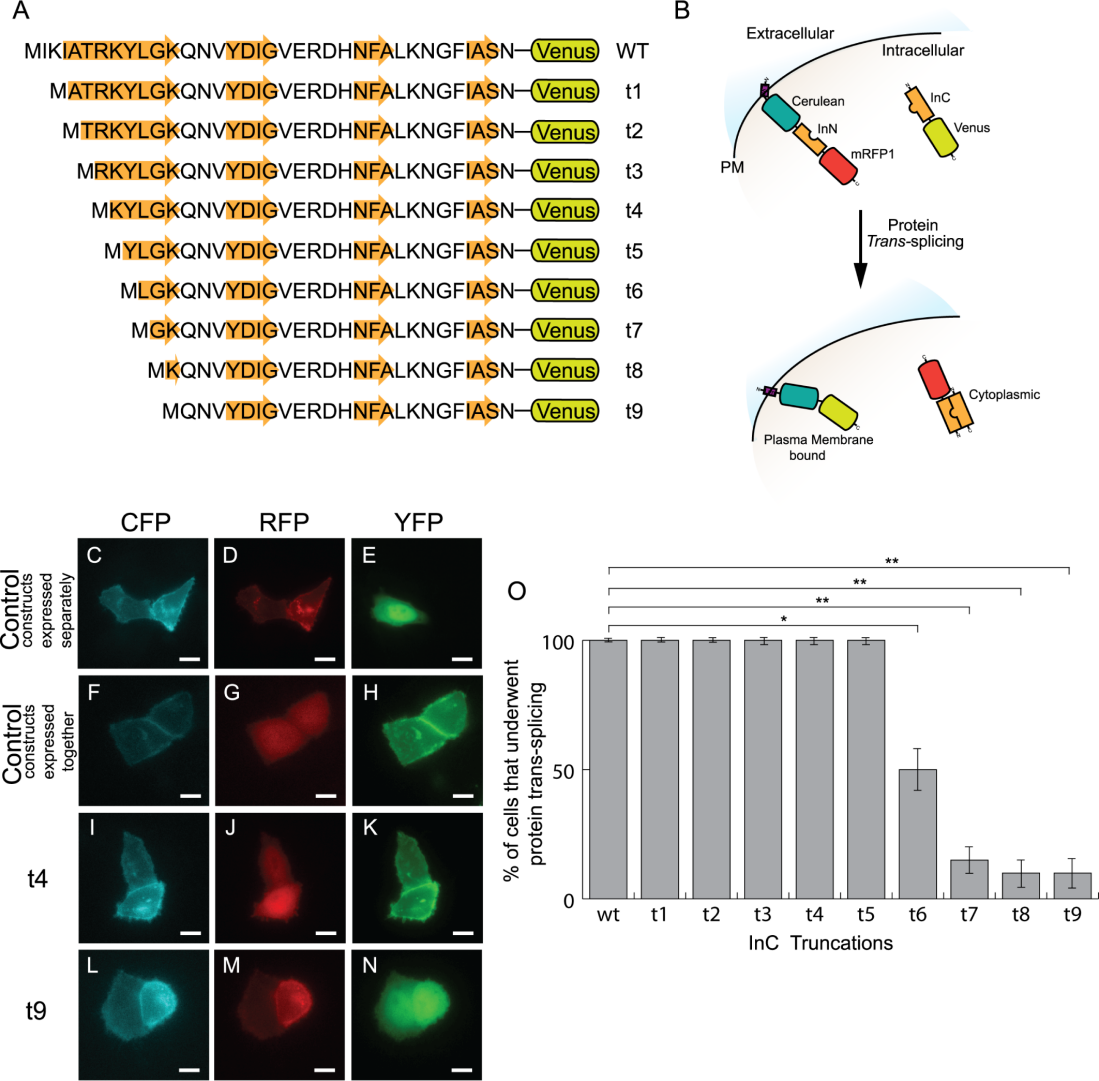
Determining the minimal functional *Npu*DnaE_C_ intein with PTS activity in mammalian cells. (A) Protein sequence of systematic truncations of the first N-terminus β-strand of *Npu*DnaE_C_ intein. (B) Schematic diagram of constructs used for establishing PTS activity, where Lyn is a plasma membrane (PM) localization sequence (shown in purple). Successful PTS activity would result in Cerulean and Venus localized to the PM while mRFP would be localized to the cytoplasm. HeLa cells expressing M-Cerulean-InN-mRFP resulted in (C) CFP and (D) RFP fluorescence localized to the PM. (D) Expression of InC-Venus resulted in YFP fluorescence distributed in the cytoplasm. Co-expression of the two constructs triggered PTS activity resulting PM localization of (F) CFP and (H) YFP while the cytoplasm is (G) RFP fluorescence. Co-expression of M-Cerulean-InN-mRFP with (I-K) t4 and (L-N) t9 truncations of N-terminus of InC in HeLa cells shown in CFP, RFP and YFP fluorescence, respectively. (O) The percentage of co-transfected cells with truncated mutants of InC (*n* = 6 experiments with over 20 cells). β-strands are shown in orange arrow. Comparison between wild-type: *p < 0.005, ** *p <* 0.001. Error bars are standard deviation. Scale bars are 10 μm. Images are in false colour.

The best location to fuse LOV2-Ja to any protein of interest is at the N-terminus because the conformational change of LOV2-Ja happens on the C-terminal end. The C-terminal fragment of *Npu*DnaE (InC) was chosen rather than the N-terminal fragment (InN). Previous studies have demonstrated that InC can accommodate long chain peptides fused to its non-splicing ends without hindering PTS activity [37,38]. It follows that the fusion of LOV2 domain (a protein of ~110 amino acids) to the N-terminus of InC would not significantly block PTS activity. Following a similar truncation strategy as performed by our group and other groups [26,28,30], we looked into truncating InC in order to maximize the allosteric effects of LOV2 on InC. Thus, the minimal functional InC intein was determined by systematically truncating the first N-terminal β-strand (Fig 1A). The efficacy of PTS activity was assayed in live cell imaging by co-expressing the C-terminal precursor comprised of a truncated InC fused to Venus (i.e. t#-InC-Venus) with the N-terminal precursor comprised of a tandem fusion of the plasma membrane (PM) localization peptide from Lyn kinase (Lyn) [39] the cyan fluorescent protein mutant Cerulean [40] InN and mRFP (i.e. M-Cerulean-InN-mRFP). The number of cells that had successfully undergone PTS activity was then counted. In the absence of PTS activity, the PM would be labeled with Cerulean and mRFP fluorescence while the cytoplasm was labeled with Venus fluorescence (Fig 1B–1D and 1L–1N). Successful PTS activity resulted in the cytoplasmic localization of mRFP and the PM localization of Cerulean and Venus (Fig 1F–1K). PTS activity was considered efficient and complete if all co-expressing cells showed the interchange between mRFP and Venus fluorescence. Otherwise, the percentage of cells that underwent PTS activity was determined (Fig 1O). As a control, HeLa cells were co-expressed with the N-terminal precursor and the wild type InC fused to Venus (i.e. wt-InC-Venus) and resulted in complete PTS activity (Fig 1F–1H and 1O). While the removal of up to 5 amino acids from the β-strand had no effect on PTS activity as all co-expressing cells underwent complete PTS activity, the removal of more than 5 amino acids significantly diminished PTS activity (*p <* 0.005) (Fig 1O). Thus, InC truncations t1-t5 was used for further studies.

### Design of LOV2 NpuDnaE intein

**Fig 2:**
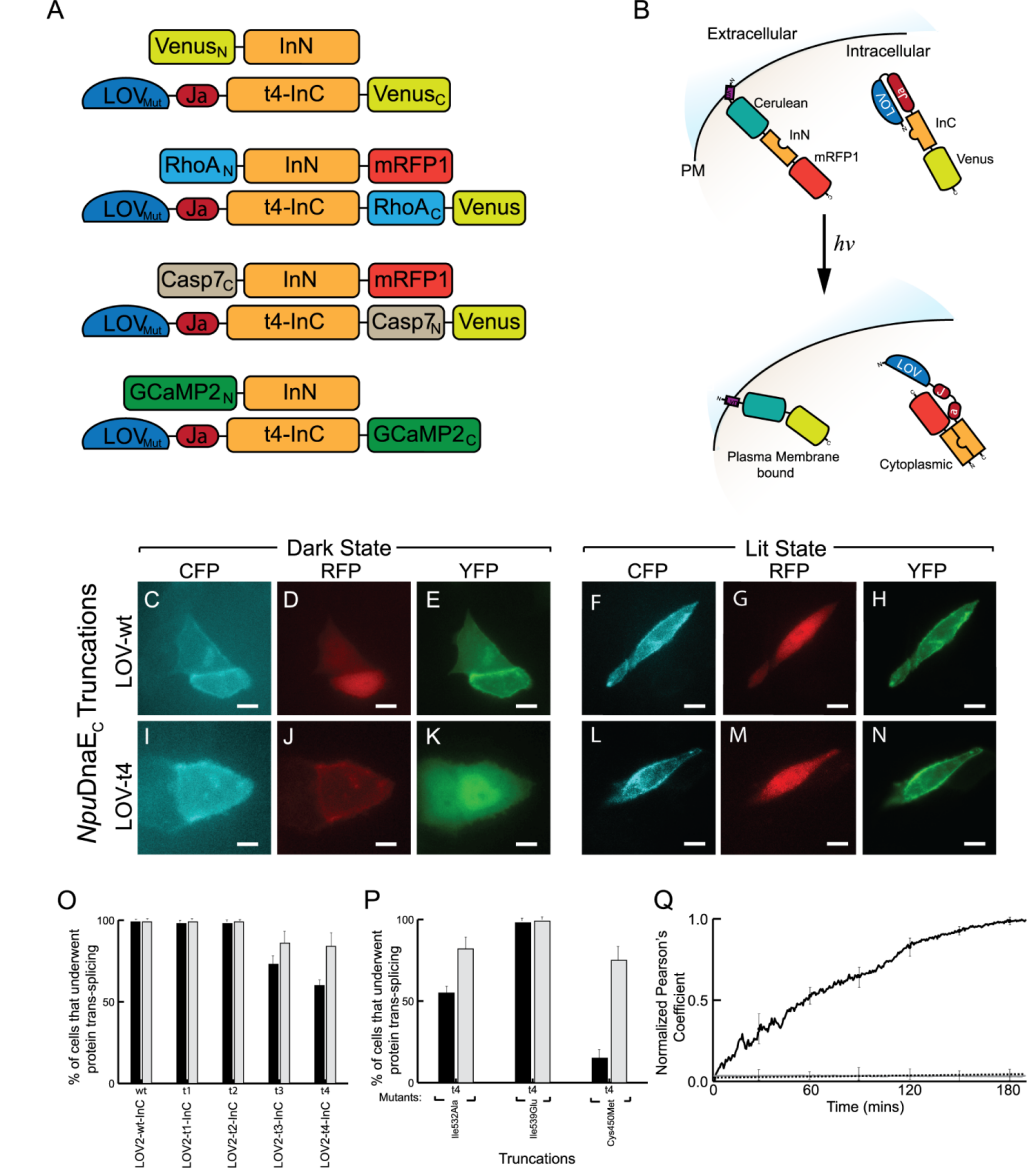
Photoactivatable *Npu*DnaE intein via LOV2 domain. (A) Layout of the different protein constructs used in this study. ‘N’ and ‘C’ denote amino and carboxyl fragments, respectively. (B) General schematic diagram of photoactivatable *Npu*DnaE intein before and after photostimulation with blue-light (Dark- and Lit-state, respectively). Blue-light photostimulation releases InC inhibition and allows protein *trans*-splicing to proceed. HeLa cells co-expressing M-Cerulean-InN-mRFP with LOV2-Jα fused to wild type (wt) InC (C-E) before and (F-H) after photostimulation. Similary, ‘t4’ truncated InC mutant co-expressed with M-Cerulean-InC-mRFP (I-K) before and (L-N) after photostimulation in HeLa cells shown in CFP, RFP, and YFP fluorescence, respectively. (O) The percentage of co-transfected cells with non-mutant LOV2 that has undergone PTS activity. (P) Percentage of co-transfected cells with mutant forms of LOV2 that has undergone PTS activity. (Q) Time course showing co-localization of CFP and YFP after blue-light photostimulation as measured by normalized Pearson’s coefficient (black trace), baseline without photostimulation (dotted line), and baseline with photostimulation of construct without LOV (grey trace). ‘N’ and ‘C’ denote amino and carboxyl fragments, respectively. Black and grey bars refer to dark- and lit- states, respectively. Error bars represent standard deviation, *n* > 6 experiments with over 20 cells observed.

In theory, a particular fusion of LOV2 to the t1-t5 truncation mutants of InC could allow the dark-state closed conformation of LOV2 to allosterically inhibit intein dimerization and subsequent PTS activity. Illumination by blue-light could induce the open conformation of LOV2 to release the inhibition of intein-dimerization and allow subsequent PTS activity (Fig 2B). As expected, live cell imaging of HeLa cells co-expressing M-Cerulean-InN-mRFP and LOV2-wt-InC-Venus (i.e. fusion with wild-type InC) expressed constitutive PTS activity in dark- and lit-states as observed by cytoplasmic localization of red fluorescence and plasma membrane localization of cyan and yellow fluorescence (Fig 2C–2H and 2O). Likewise, fusion of LOV2 to InC truncations t1 or t2 had no effect on PTS activity irrespective of photostimulation. On the other hand, fusion of LOV2 to InC truncations t3 or t4 had an increase of PTS activity between the dark- and lit-states with the greatest change occurring in truncations t4 (~40% increase in PTS activity from dark- to lit-state; *n* = 6) (Fig 2I–2N and 2O), whereas truncation t3 was only minimally affected (~18% increase in PTS activity; *n* = 6). Furthermore, fusion of LOV2 to InC truncation t5 abolished PTS activity. Thus, InC truncation with t4 was further investigated.

### LOV2 mutants improve the photoactivatable NpuDnaE intein

Initially, LOV2 was replaced with the LOV2_I532A_ mutant as it has been shown to reduce dark-state activation by 75% in other designs [25]. However co-expression of LOV2_I532A_-t4*Npu*DnaE_C_-Venus with M-Cerulean-InN-mRFP did not further suppress dark-state PTS activity nor did it increase the change between dark- and lit-state PTS activity (~49% increase in PTS activity from dark- to lit-state; *n* = 6) (Fig 2P). Next, we used the C450M mutant to achieve a suppression of the dark-state PTS activity, while allowing enhanced activity in the light-state when exposed to light for long durations (i.e. greater than 4 hours). At first, this may seem counter-intuitive because the C450M is known to interfere with covalent adduct formation, resulting in a dark-state preference by promoting LOV2 docking of the Jα helix [28,41]. However, nuclear magnetic resonance (NMR) studies showed that exposure to light, in the time scale of hours, lead to irreversible adduct formation as evident from the appearance of a specific triplet chemical shift, indicating an interaction between the sulphur atom of methionine and the flavin mononucleotide (FMN) radical at the N(5) position [42,43]. This irreversible adduct formation results in a permanent lit-state conformation where LOV2 cannot dock again to the Jα helix. Accordingly in this study, after replacing the LOV2 domain with LOV2_C450M_ to yield LOV2_C450M_-t4-InC-Venus, HeLa cells were co-transfected with M-Cerulean-InN-mRFP and LOV2_C450M_-t4-InC-Venus. As expected, there was diminished PTS activity in the absence of light but a four-fold increase in PTS activity when photo-stimulated with blue-light for over 4 hours (Fig 2P). As a control, the LOV2_I539E_ mutant was used to confer a lit-state preference by promoting LOV2 undocking of the Jα helix [28,41]. Accordingly, LOV2_I539E_ fused to t4-InC-Venus and co-transfected with M-Cerulean-InN-mRFP in HeLa cells underwent complete PTS activity irrespective of photostimulation (Fig 2P).

Next using this LOV2_C450M_-t4-InC-Venus mutant, the kinetics of protein *trans*-splicing translocation in single live cells was determined by measuring the Pearson’s coefficient of Cerulean and Venus fluorescence over time. This revealed significant protein *trans*-splicing occurred at approximately 120±23 mins (*n* = 6) after the start of photo-stimulation (Fig 2Q). Lastly, a fluorescent SDS-PAGE analysis revealed the formation of spliced products from its precursors after blue-light photostimulation (S4 Fig). Due to the increased photodynamic range of this LOV_C450M_-t4-InC construct (hereafter, LOVInC), it was further used to engineer photoactivated reassembly of target proteins such as Venus, RhoA, Caspase-7, and GCaMP2 (Fig 2A).

### Photoactivatable reassembly of Venus mediated by LOVInC

**Fig 3:**
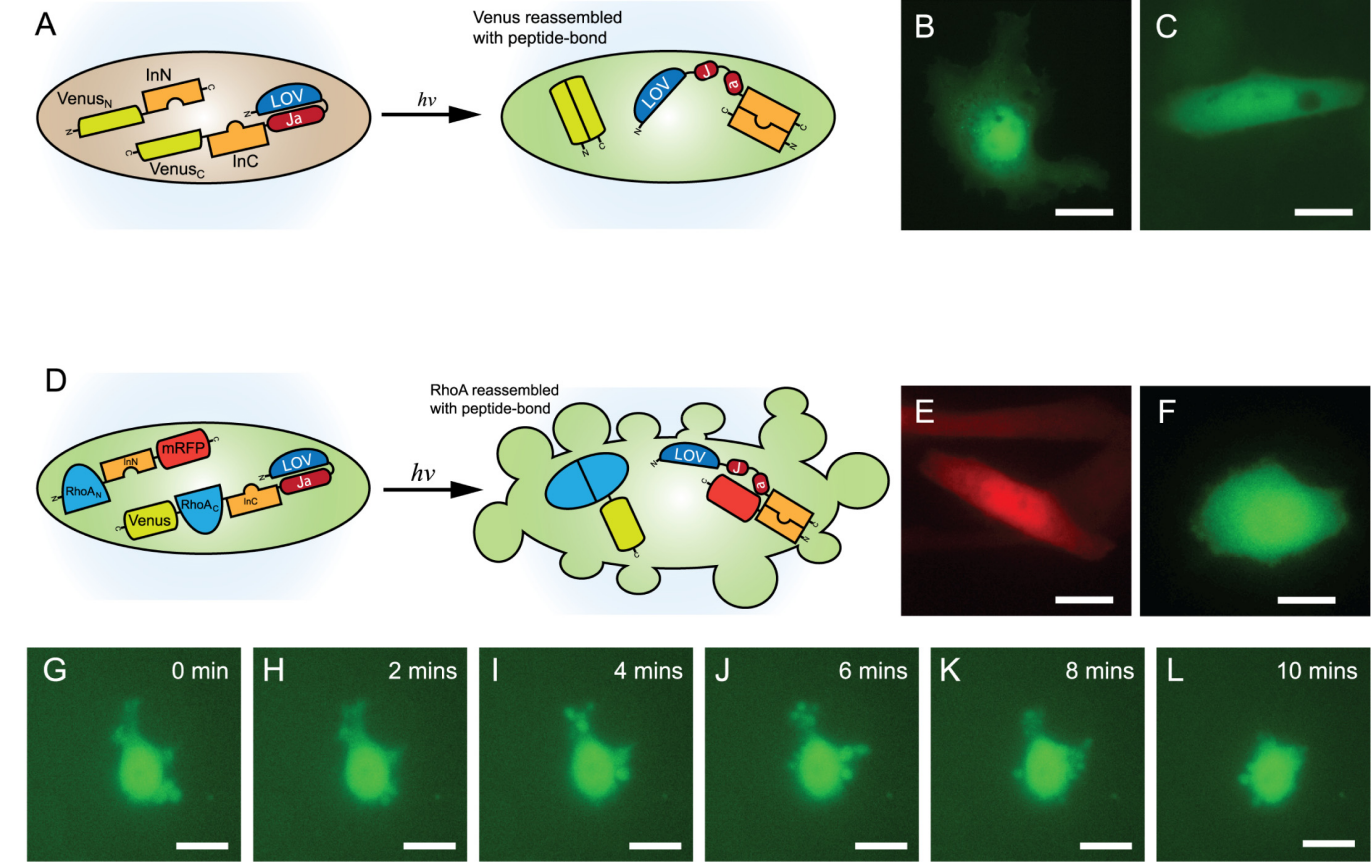
Photoactivatable reassembly of split Venus fluorescence and split RhoA. (A) General schematic diagram of reassembly of split Venus fluorescence after blue-light photostimulation. (B) HeLa cells co-expressing Venus_N_-InN and LOVInC-Venus_C_ showing the restoration of YFP fluorescence after photostimulation with blue-light. (C) HeLa co-expressing split Venus fused to its respective intein fragment as control. (D) General schematic diagram of reassembly of split RhoA by photoactivatable PTS activity. Expression of (E) RhoA_N_-InN-mRFP or (F) LOVInC-RhoA_C_-Venus alone did not result in continuous dynamic blebbing. (G-L) However, co-expression of the two constructs resulted in continuous dynamic blebbing in HeLa cells stemming from the functional reassembled RhoA after photostimulated PTS activity (*n* = 10 experiments). Scale bars are 10 μm. Images are in false colour.

The C-terminal construct comprised of a tandem fusion LOVInC to Venus_C_ (fragment containing residues 145–238) was co-transfected in mammalian cells with the N-terminal construct comprised of Venus_N_ (1–144) tandemly fused to InN (Venus_N_-InN) (Fig 3A). Overnight photostimulation yielded cells with Venus fluorescence similar to the *Npu*DnaE reassembly of split Venus without the LOV2 domain (Fig 3B and 3C, respectively) (*n* = 6). In contrast, co-expressing cells grown in the absence of light exhibited no detectable fluorescence (*n* = 6). Further, expressions of either the N- or C-terminal constructs alone did not produce any fluorescence (*n* = 6). Lastly, a fluorescent SDS-PAGE analysis revealed the formation of spliced products from its precursors after blue-light photostimulation (S1 Fig).

### Photoactivatable reassembly of RhoA mediated by LOVInC

RhoA is a member of the Rho GTPase family of small molecular switches important in regulating cell morphology and motility [28,44,45]. In particular, when the dominant positive RhoA (Q63L) is expressed in certain epithelial cells (e.g. Hela cells), the cells exhibited dynamic and non-apoptotic blebbing [34,46,47]. The C-terminal construct was prepared by the tandem fusion of LOVInC, RhoA_C_ (1–50), and Venus (i.e. LOVInC-RhoA_C_-Venus) while the N-terminal construct was prepared by tandem fusing RhoA_N_ (51–193) to InN and mRFP (i.e. RhoA_N_-InN-mRFP) (Fig 3D). Cells expressing either constructs alone did not undergo dynamic blebbing (Fig 3E and 3F, respectively) (*n* = 6). However, when LOVInC-RhoA_C_-Venus and RhoA_N_-InN-mRFP were co-transfected in HeLa cells and photostimulated overnight, co-expressing cells underwent dynamic blebbing (Fig 3G–3L and S1 Video). Cells grown in the absence of blue light did not undergo dynamic blebbing (*n* = 6). Lastly, a fluorescent SDS-PAGE analysis revealed the formation of spliced products from its precursors after blue-light photostimulation (S2 Fig).

### Photoactivatable reassembly of Caspase-7 mediated by LOVInC

**Fig 4:**
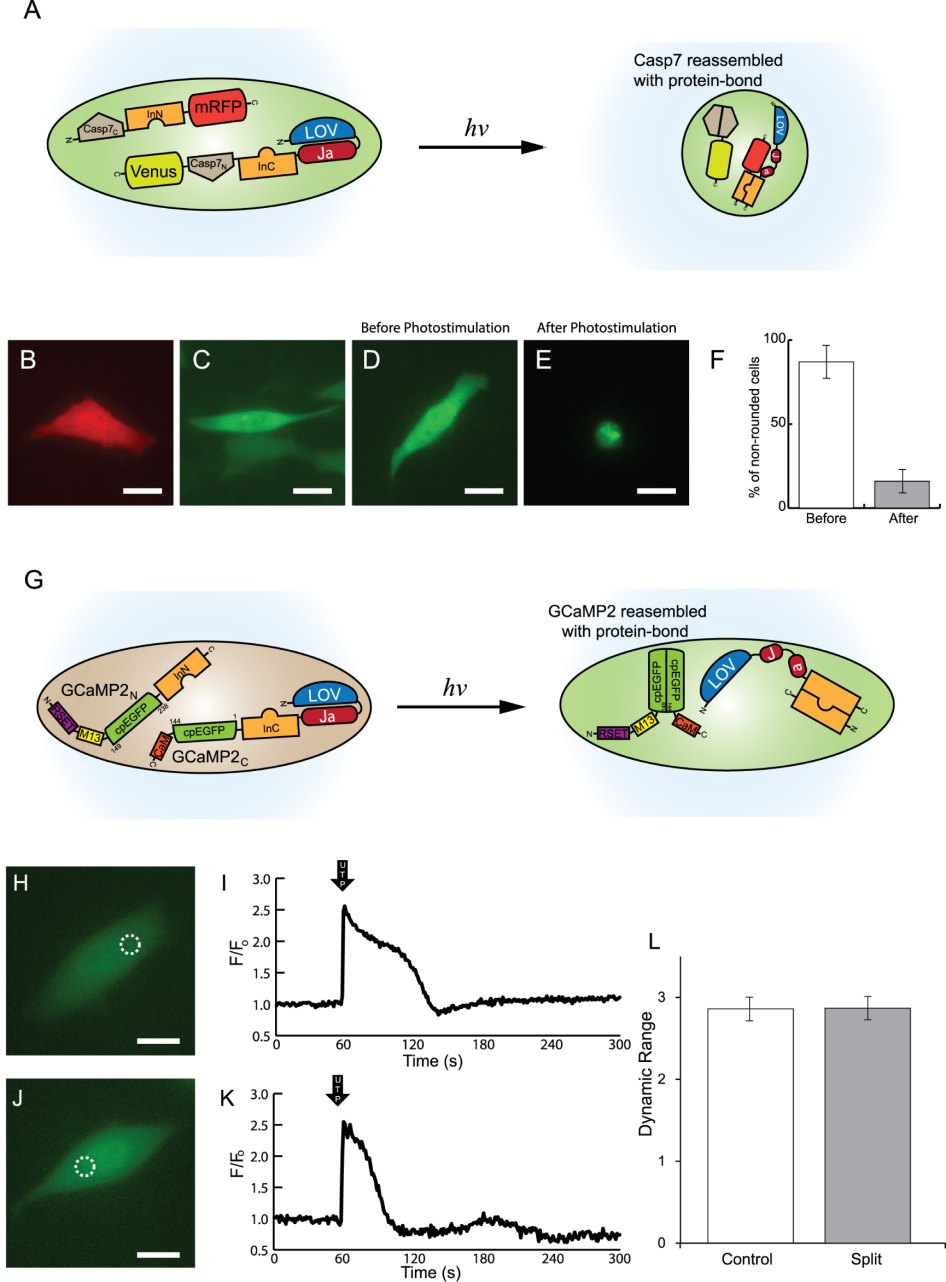
Photoactivatable reassembly of split caspase-7 and split GCaMP2 Ca^2+^ biosensor. (A) General schematic diagram of reassembly of split caspase-7 by photoactivated PTS activity. Expression of (B) Casp7_C_-InN-mRFP or (C) LOVInC-Casp7_N_-Venus alone did not result in apoptotic-like morphological changes. However, HeLa cells co-transfected with Casp7_C_-InN-mRFP and LOVInC-Casp7_N_-Venus did not express apoptotic-like morphological rounding (D) before photostimulation, but did (E) after photostimulation. (F) Percentage of non-rounded cells before and after photostimulation (*n* = 6 experiments with over 20 cells). (G) General schematic diagram of reassembly of split GaMP2 biosensor by photoactivatable PTS activity. (H) YFP fluorescence image of HeLa cells co-expressing GCaMP2_N_-InN and LOVInC-GCaMP2_C_ and its (I) UTP-induced Ca^2+^ transient as measured by the reassembled GCaMP2 biosensor. (J) YFP fluorescence image of HeLa cell expressing control GCaMP2 biosensor and its (K) measured UTP-induced Ca^2+^ transient. (L) Comparison of mean dynamic range of control and reassembled GCaMP2 (*n* = 6 experiments). Scale bars are 10 μm. Dotted circle represent regions where average fluorescence intensity measurements were taken. Images are in false colour.

Caspase-7 (hereafter, Casp7) is a member of a conserved family of tightly-regulated proteases whose activation results in the dismantling of the cell machinery and the irreversible commitment to apoptosis, or programmed cell death [48,49]. When a prodomain is cleaved from inactive Casp7 by upstream caspases, it undergoes auto-proteolysis into a p20 subunit (fragment containing residues 57–198) and p11 subunit (207–303) that associate to form an active Casp7 [48]. By mimicking these proteolytic events, a constitutively active Casp7 was engineered by creating a tandem fusion of Casp7_C_ (p11 subunit) and Casp7_N_ (p20 subunit) [50]. The N-terminal construct was composed of a tandem fusion of Casp7_C_, *Npu*DnaE_N_, and mRFP (i.e. Casp7_C_-InN-mRFP) while the C-terminal construct was comprised of a tandem fusion of LOV_C450M_, InC, Casp7_N_, and Venus (i.e. LOVInC-Casp7_N_-Venus) (Fig 4A). Cells expressing each of the constructs separately did not exhibit any morphological changes when photostimulated with blue light (Fig 4B and 4C, respectively) (*n* = 6). HeLa cells co-transfected with the two constructs and photostimulated with blue-light induced morphological changes (87.3 ± 6%) mimicking that of apoptosis such as the shrinking and rounding of cells and loss of nuclear envelope integrity that were similar to a control of active Casp7 (Fig 4D and 4F). Co-transfected cells grown in the absence of photostimulation did not undergo morphological changes (16.7 ± 7%) (Fig 4E and 4F). Lastly, a fluorescent SDS-PAGE analysis revealed the formation of spliced products from its precursors after blue-light photostimulation (S3 Fig).

### Photoactivatable reassembly of GCaMP2 mediated by LOVInC

GCaMP2 is a commonly used single fluorophore intensity-based Ca^2+^ biosensor composed of a tandem fusion of a calmodulin binding peptide from myosin light chain kinase (M13), a circularly permutated EGFP (cpEGFP), and calmodulin [35,36]. GCaMP2 was split at the junction of circular permutation (i.e. GCaMP2_N_ and GCaMP2_C_) as previously described [37]. The C-terminal construct was composed of a tandem fusion of LOVInC and GCaMP2_C_ (i.e. LOVInC-GCaMP2_C_) while the N-terminal construct was a tandem fusion of GCaMP2_N_ and InN (i.e. GCaMP2_N_-InN) (Fig 4G). HeLa cells were then co-transfected with the two constructs and grown overnight in the presence of periodic blue-light photostimulation. UTP-induced Ca^2+^ transients were measured via the reassembled GCaMP2 biosensor (Fig 4H) and compared to the native GCaMP2 biosensor (Fig 4J). The reassembled GCaMP2 biosensor had similar measured Ca^2+^ transient profiles (Fig 4I and 4K, respectively) and statistically indistinguishable dynamic range compared to that of the native GCaMP2 biosensor (2.87 ± 0.18 and 2.85 ± 0.16, respectively; *p* = 0.98, *n* > 6) (Fig 4L). As expected, co-transfected cells grown in the absence of blue-light photostimulation did not yield any reassembled GCaMP2 biosensor and UTP-induced Ca^2+^ transients could not be measured. In addition, cells expressing only GCaMP2_N_-InN or LOVInC-GCaMP2_C_ did not display any GCaMP2 activity or fluorescence.

### Spatial and temporal control of protein trans-splicing mediated by LOVInC

**Fig 5:**
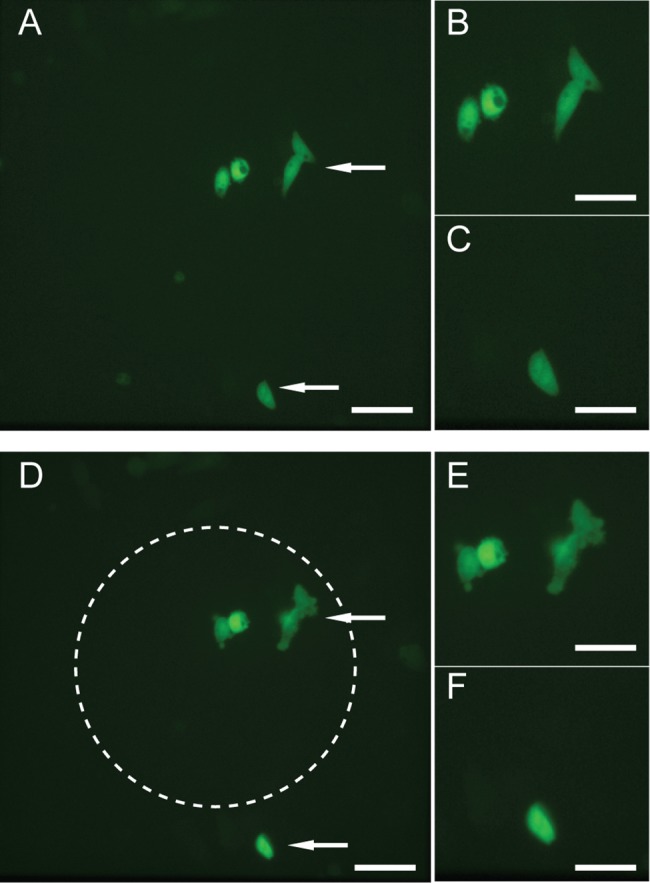
Photoactivatable intein has spatial precision. (A) HeLa cells co-expressing RhoA_N_-InN-mRFP and LOVInC-RhoA_C_-Venus and viewed under low magnification (20x objective). Two groups of cells that have been identified by white arrows and shown enlarged in panels B and C. Cells located near the center of the field were photostimulated with periodic interval of blue-light (1 second every 0.5 min). (D) Low magnification of the same set of cells after 150 mins of showing cells within the illumination zone (dotted white circle) have undergone dynamic blebbing while cells outside the illumination zone generally remained unchanged. Again, the two groups of cells are shown enlarged in panels E and F. Scale bars are 50 μm for A and D and 30 μm for B, C, E, and F. Images are in false colour.

Using the LOVInC-RhoA_C_-Venus and RhoA_N_-InN-mRFP constructs as example, protein trans-splicing was restricted to an area defined by light. A narrow beam of blue-light was used to periodically illuminate (i.e. 1 s exposure every 30 s) a field of HeLa cells co-expressing the constructs LOVInC-RhoA_C_-Venus and RhoA_N_-InN-mRFP (Fig 5A–5C). After approximately 150 minutes, cells situated within the illuminated central region showed dynamic blebbing (81 ± 10%), while cells located outside remained unchanged (18 ± 7%) (Fig 5D–5F).

The temporal control of PTS in our LOVInC system had a slower kinetic activity of PTS than wild-type Npu DnaE intein. The Npu DnaE intein is one of the fastest inteins known with a response time in the time scale of minutes [32], while our LOVInC system has a response time in the time scale of hours. Since the C-Intein fragment was gradually truncated to the point where it began to lose activity, it is likely that the kinetic activity was also compromised. Furthermore, although the LOV2-Ja in the lit state releases Ja peptide, the bulky LOV domain could still affect the accessibility for the C-intein active site for the N-intein. Lastly, the target protein that is reformed from the protein splicing needs to re-fold before its active, further leading to a slower response time.

### Concluding remarks

PTS activity is a post-translational modification that is facilitated by inteins. All PTS activity occurs spontaneously once the precursors are expressed, ligating the two corresponding extein peptide fragments together with a native peptide bond. As such, the PTS activity can be exploited as a means to restore the function of split target proteins. The applications of protein splicing can be expanded by mechanisms that can adequately control PTS activities. *Cis*-splicing inteins have been artificially split to generate precursor fragments that do not self-associate. By fusing these precursors to exogenous binding pairs, activity can be regulated by controlling the association of the binding pairs [21,51]. However, the precursors are often limited by their reduced solubility and protein splicing efficiencies, especially when fused to non-native exteins. On the other hand, the naturally split inteins have better solubility and splice with higher efficiencies, particularly when fused to heterologous protein fragments [11]. Using chemical caging, PTS activity of naturally split inteins has been abolished by disrupting the splice sites or preventing formation of secondary structures required for intein association [14,15]. However, the generation of the precursors require *ex vivo* protein synthesis and modification.

The advantage of light as a control mechanism is its spatial and temporal precision coupled with minimal phototoxicity at moderate dosages. Unlike other previous approaches, our strategy does not require *ex vivo* protein synthesis or exogenous co-factors. Repeated photo-stimulation of LOVInC induced protein *trans*-splicing to reassemble and restore function to several split target proteins in mammalian cells. In addition, LOVInC exhibited spatiotemporal precision by triggering protein *trans*-splicing in selected photo-stimulated cells. The modularity of LOVInC allows tagging to a variety of target proteins using common genetic manipulations. It should be noted that there may be a possibility that protein concentration may have an effect on protein splicing. Since the LOV2 domain is used in an allosteric manner to decrease the affinity of the intein fragments, one can expect that in situations with very high protein expression levels, PTS may occur in the absence of photostimulation as the high protein concentration may overcome the decreased intein fragment affinity.

Using the LOVInC system, we were able to engineered a synthetic split intein bio-system controlled by light. We then tested this system to show photo-regulation of a variety of target proteins–Venus, RhoA, GCaMP2 and Caspase-7 –that may have applications in tissue specific expression guided by both orthogonal promoters and light. The use of a single tissue specific promoter often label cells beyond those of interest, and finding completely specific promoters is a non-trivial task [52]. The use of two orthogonal promoters can provide increased tissue specificity with existing promoter libraries and the combination with light can offer further targeting to sub-tissues of interests. For example, the LOVInC system with Venus can be used like photoactivable fluorescent proteins to track cell migration of labeled cells, but when coupled with orthogonal promoters, it allows tracking of specific cells within a targeted tissue. Likewise, the LOVInC system with caspase-7 can be used like a photoactivated caspase-7 [30], but with greater specificity to study the effects within an organism from the death of particular cells at a targeted tissue. The LOVInC system with GCaMP2 can be specifically targeted to individual neurons to image Ca^2+^ signals for mapping neural circuits. Unlike the previous photoactivable intein designs that required *ex vivo* protein synthesis, LOVInC is genetically encoded and can be easily expressed in biological systems by genetic means and thus, it may find wider applicability in transgenic model organisms. In transgenic models, we envision proteins such as LOVInC-GCaMP2_C_ and GCaMP2_N_-InN can be expressed by two orthogonal tissue-specific promoters to allow Ca^2+^ imaging in specific cell populations. Furthermore, when coupled with the spatiotemporal precision offered by light, it can provide control over when and where to express a protein in a transgenic organism.

## Experimental Procedures

### Plasmid construct and subcloning

Gene fragments were amplified from plasmid or cDNA sources and subcloned into pCfVtx3 as previously described [53,54]. InN, InC, RhoA(DP), and LOV2 were cloned from Addgene plasmids 12172, 15335, 12968, and 22027. The catalytic domain of caspase-7 was cloned from human cDNA. The first three amino acids of the native extein sequence of InC were included. N- and C- terminal portions of split Venus, RhoA, caspase-7, and GCaMP2 were obtained and amplified by standard PCR methods. The particular fragments selected for splitting were chosen because they are supported by previous studies that show they are good locations. For instance, in the Venus case, the Venus fragments (145–238 and 1–144) were derived from the pericam Ca^2+^ biosensor design as it was the site of circular permutation of YFP [55]. In the case of caspase-7, caspase fragments (57–198 and 207–303) are naturally produced from self-cleavage and the circular permutation of these fragments results in an active caspase [48]. In the case of RhoA, RhoA fragments (1–50 and 51–193) were derived from our design of a Ca^2+^-sensitive RhoA as it was the site of insertion of the calmodulin binding site [34].

### Cell culture and transfection

HeLa cell line (from ATCC; RR-B51S(PTS-5258)) was maintained in Dulbecco’s Modified Eagle’s Medium (DMEM) supplemented with 25 mM D-glucose, 1 mM sodium pyruvate, 4mM L-glutamine (Invitrogen, Carlsbad, USA), 10% FBS (Invitrogen), and 10 mL/L penicillin-streptomycin solution (Sigma-Aldrich, St. Louis, USA) in T5 flasks at 37°C and 5% CO_2_. Cells were grown to 95% confluency before passaging with 0.05% trypsin-EDTA (Sigma-Aldrich). Passaged cells were seeded onto glass-bottom dishes (MatTek, Ashland, MA) at 1:10 dilution and grown overnight. Cells were transiently transfected with plasmids and Lipofectamine 2000 according to manufacturer’s protocols (Invitrogen).

### Imaging and illumination

All cell imaging were performed in DMEM media except for Ca^2+^ transient measurements. Imaging was performed using an inverted IX81 non-confocal microscope with a Lambda DG4 xenon lamp source and QuantEM 512SC CCD camera (Olympus). Excitation and emission filter specifications were as follows: CFP (Ex: 438/24 nm; Em: 482/32 nm); YFP (Ex: 500/24 nm; Em: 542/27 nm); and RFP (Ex: 580/20 nm; Em: 630/60 nm) (Semrock). Unless otherwise stated, the excitation of LOV2 was conducted by periodically illuminating samples overnight for 1 second at 30 second intervals using CFP filter. The light intensity was 25 mW/cm^2^. For Ca^2+^ transient experiments, cell culture media were replaced with PBS supplemented with 5 mM CaCl_2_ and 1 mM MgCl_2_ (Invitrogen).

### Statistical Analysis

For light simulation experiments, data is presented as the mean ± standard deviation with at least 6 independent experiments with at least 20 cells. In cases where cells were observed, *n* = 6 experiments each with more than 20 cells were observed unless otherwise noted. Splicing levels were determined by calculating the Pearson’s coefficient (PC) of the co-localization of Cerulean and Venus fluorescent proteins under CFP and YFP fluorescent filters, respectively. A threshold level of PC≥0.95 was set such that cells exhibiting PC values equal or above 0.95 were counted as having undergone PTS activity while those that fall below 0.95 did not undergo PTS activity. Normalized Pearson’s coefficient was determined by subtracting the minimum value from the data points and then normalizing the resulting points against the maximum value. GCaMP2 fluorescence intensity measurements are reported as *F/F*_*o*_, were *F* is the raw fluorescence intensity time series minus background fluorescence and *F*_*o*_ is the mean fluorescence signal during the baseline period prior to Ca^2+^ transient stimulations. Significance between conditions was calculated using Student’s *t*-test and *p*<0.05 were considered statistically significant.

## Supporting Information

S1 Fig Fluorescent SDS-PAGE before and after photo-induced PTS for the reassembly of split Venus. Protein precursors were expressed in *E*. *coli* and either grown in the dark (i.e. absence of photostimulation) or grown in the presence of overnight periodic blue-light photostimulation (1 s every 30 s interval). Proteins were extracted by sonication and separated on SDS-PAGE. Fluorescent SDS-PAGE gel separation of constructs expressed separately (Lanes 1 and 2), together in the dark state (Lane 3) and lit state (Lane 4). The reassembled split Venus can be observed after photostimulation. Lane 1: Venus_N_-InN; Lane 2: LOVInC-Venus_C_; Lane 3: Co-expression of Venus_N_-InN and LOVInC-Venus_C_ before photostimulation; Lane 4: Co-expression of Venus_N_-InN and LOVInC-Venus_C_ after photostimulation.
(TIFF)

S2 Fig Fluorescent SDS-PAGE before and after photo-induced PTS for the reassembly of split RhoA(dp). Protein precursors were expressed in *E*. *coli* and either grown in the dark (i.e. absence of photostimulation) or grown in the presence of overnight periodic blue-light photostimulation (1 s every 30 s interval). Proteins were extracted by sonication and separated on SDS-PAGE. Fluorescent SDS-PAGE gel separation of constructs expressed separately (Lanes 1 and 2), together in the dark state (Lane 3) and lit state(Lane 4). The reassembled RhoA was formed after photostimulation (Lane 4). The faint red bands at ~58 kDa and ~48 kDa in Lanes 2, 3, and 4 under the red fluorescence filter is the result of fluorescence bleed-through from the Venus. Lane 1: RhoA_N_-InN-mRFP; Lane 2: LOVInC-RhoA_C_-Venus; Lane 3: Co-expression of RhoA_N_-InN-mRFP and LOVInC-RhoA_C_-Venus before photostimulation; Lane 4: Co-expression of RhoA_N_-InN-mRFP and LOVInC-RhoA_C_-Venus after photostimulation.
(TIFF)

S3 Fig Fluorescent SDS-PAGE before and after photo-induced PTS for the reassembly of split caspase-7. Protein precursors were expressed in *E*. *coli* and either grown in the dark (i.e. absence of photostimulation) or grown in the presence of overnight periodic blue-light photostimulation (1 s every 30 s interval). Proteins were extracted by sonication and separated on SDS-PAGE. Fluorescent SDS-PAGE gel separation of constructs expressed separately (Lanes 1 and 2), together in the dark state (Lane 3) and lit state (Lane 4). The reassembled caspase-7 was reformed after photostimulation (Lane 4). The faint red bands at ~61 kDa and ~56 kDa in Lanes 2, 3, and 4 under the red fluorescence filter is the result of fluorescence bleed-through from the Venus. Lane 1: Casp7_C_-InN-mRFP; Lane 2: LOVInC-Casp7_N_-Venus; Lane 3: Co-expression of Casp7_C_-InN-mRFP and LOVInC-Casp7_N_-Venus before photostimulation; Lane 4: Co-expression of Casp7_C_-InN-mRFP and LOVInC-Casp7_N_-Venus after photostimulation.
(TIFF)

S4 Fig Fluorescent SDS-PAGE before and after photo-induced protein *trans*-splicing. Protein precursors were expressed in *E*. *coli* and either grown in the dark (i.e. absence of photostimulation) or grown in the presence of overnight periodic blue-light photostimulation (1 s every 30 s interval). Proteins were extracted by sonication and separated on SDS-PAGE. Fluorescent SDS-PAGE gel separation of constructs expressed separately (Lanes 1 and 2), together in the dark state (Lane 3) and lit state (Lane 4) photo-induced protein *trans*-splicing. The red band at ~40 kDa in Lanes 1 and 2 under the red fluorescence filter is the result of fluorescence bleed-through from the Venus. Lane 1: M-Cerulean-InN-mRFP; Lane 2: LOVInC-Venus; Lane 3: Co-expression of M-Cerulean-InN-mRFP and LOVInC-Venus before photostimulation; Lane 4: Co-expression of M-Cerulean-InN-mRFP and LOVInC-Venus after photostimulation to yield Lyn-Cerulean-Venus and mRFP-InN.
(TIFF)

S1 Video Photoactivatable reassembly of split RhoA producing a dynamic blebbing phenotype. Co-expression of RhoA_N_-InN-mRFP and LOVInC-RhoA_C_-Venus resulted in continuous dynamic blebbing in HeLa cells stemming from the functional reassembled RhoA after photostimulated PTS activity (*n* = 10 experiments). Each second in the video corresponds to 1 minute. The video is in false colour.
(AVI)
