# Supplementary figures and images for: An Engineered Split Intein for Photoactivated Protein Trans-Splicing
Source: PLoS One. 2015 Aug 28;10(8):e0135965. doi: 10.1371/journal.pone.0135965 (PMC4552755; doi:10.1371/journal.pone.0135965)

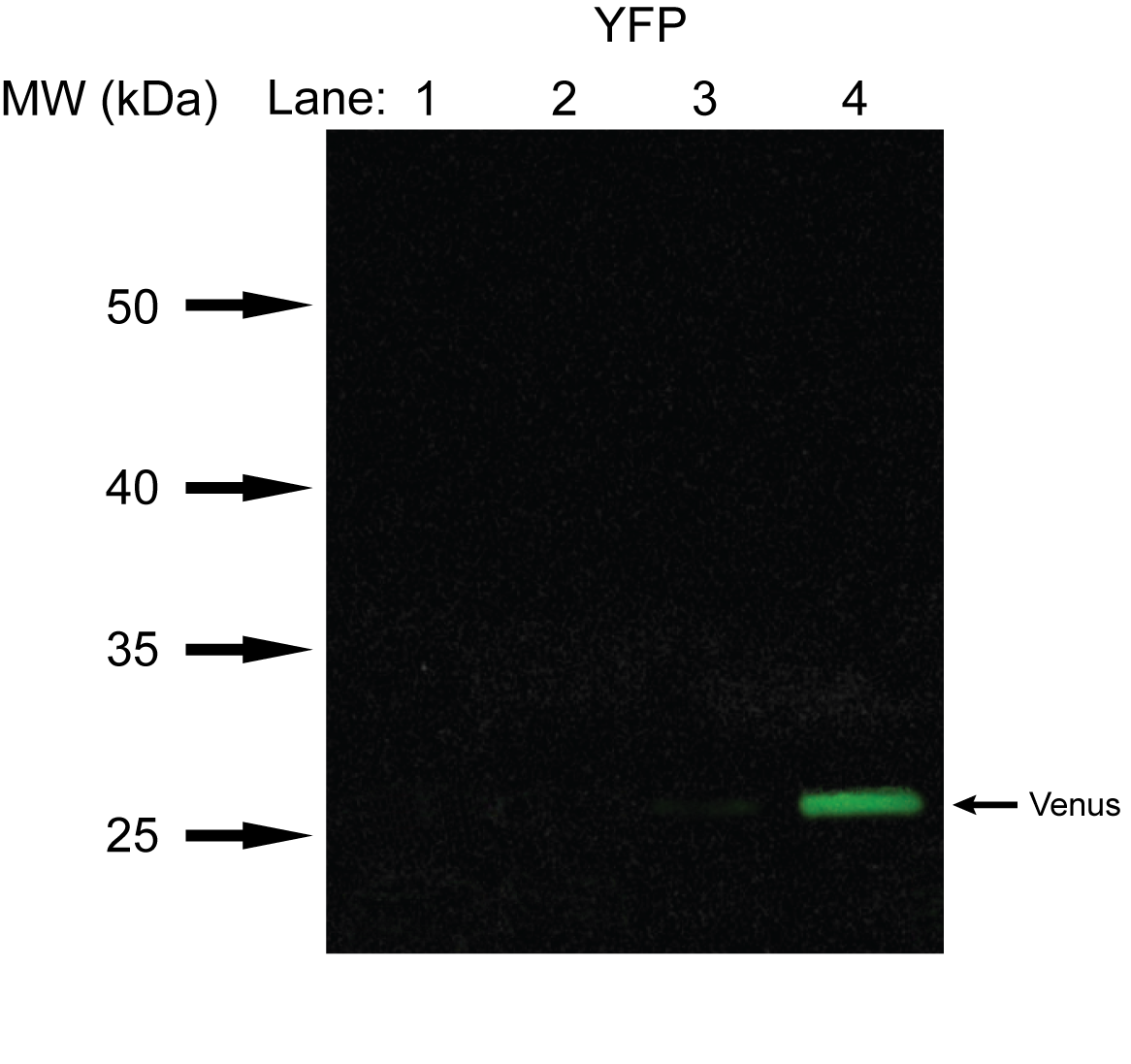

Supplement: S1 Fig — Protein precursors were expressed in E. coli and either grown in the dark (i.e. absence of photostimulation) or grown in the presence of overnight periodic blue-light photostimulation (1 s every 30 s interval). Proteins were extracted by sonication and separated on SDS-PAGE. Fluorescent SDS-PAGE gel separation of constructs expressed separately (Lanes 1 and 2), together in the dark state (Lane 3) and lit state (Lane 4). The reassembled split Venus can be observed after photostimulation. Lane 1: VenusN-InN; Lane 2: LOVInC-VenusC; Lane 3: Co-expression of VenusN-InN and LOVInC-VenusC before photostimulation; Lane 4: Co-expression of VenusN-InN and LOVInC-VenusC after photostimulation. (TIFF) [file pone.0135965.s001.tiff]

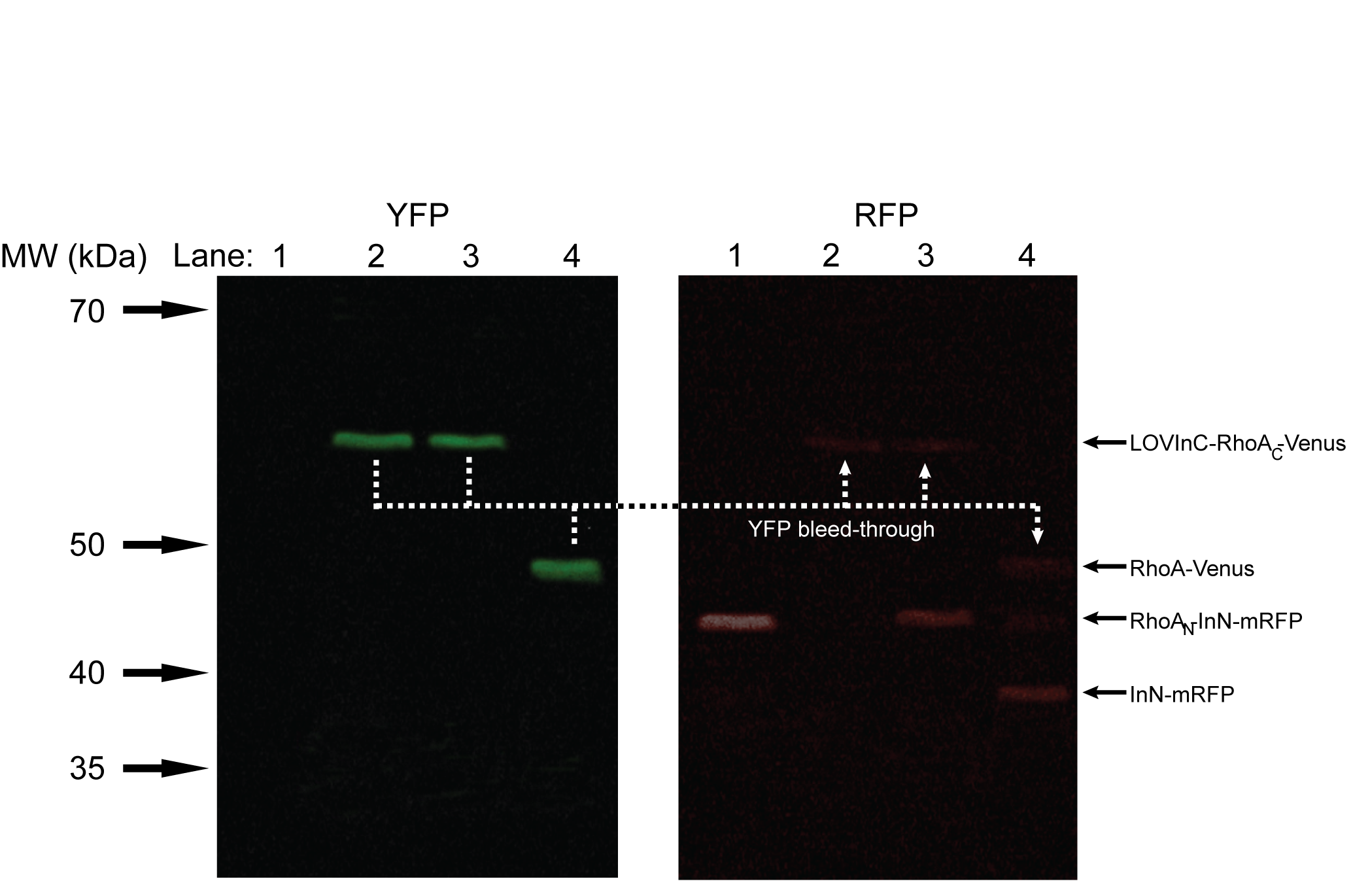

Supplement: S2 Fig — Protein precursors were expressed in E. coli and either grown in the dark (i.e. absence of photostimulation) or grown in the presence of overnight periodic blue-light photostimulation (1 s every 30 s interval). Proteins were extracted by sonication and separated on SDS-PAGE. Fluorescent SDS-PAGE gel separation of constructs expressed separately (Lanes 1 and 2), together in the dark state (Lane 3) and lit state(Lane 4). The reassembled RhoA was formed after photostimulation (Lane 4). The faint red bands at ~58 kDa and ~48 kDa in Lanes 2, 3, and 4 under the red fluorescence filter is the result of fluorescence bleed-through from the Venus. Lane 1: RhoAN-InN-mRFP; Lane 2: LOVInC-RhoAC-Venus; Lane 3: Co-expression of RhoAN-InN-mRFP and LOVInC-RhoAC-Venus before photostimulation; Lane 4: Co-expression of RhoAN-InN-mRFP and LOVInC-RhoAC-Venus after photostimulation. (TIFF) [file pone.0135965.s002.tiff]

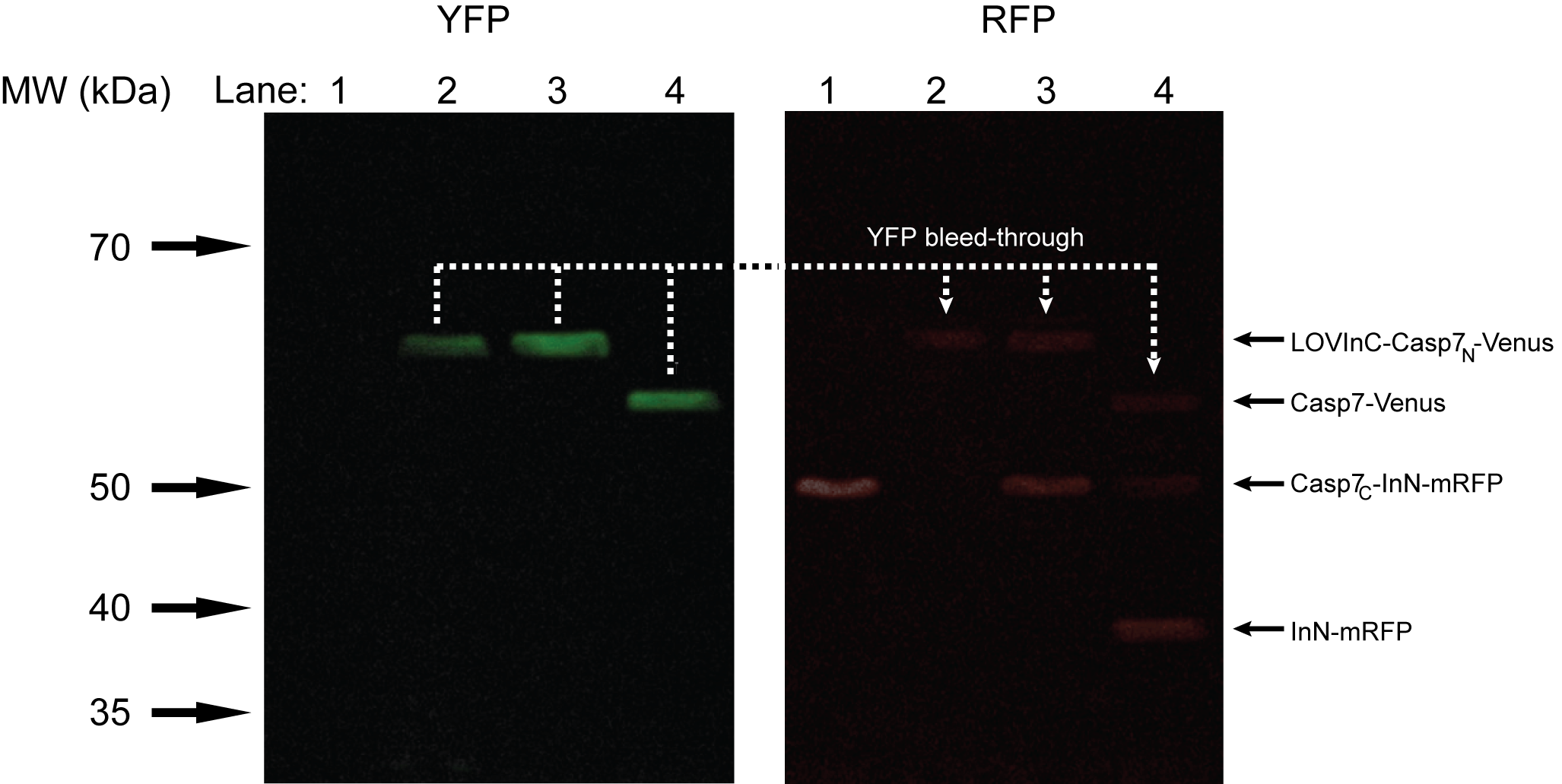

Supplement: S3 Fig — Protein precursors were expressed in E. coli and either grown in the dark (i.e. absence of photostimulation) or grown in the presence of overnight periodic blue-light photostimulation (1 s every 30 s interval). Proteins were extracted by sonication and separated on SDS-PAGE. Fluorescent SDS-PAGE gel separation of constructs expressed separately (Lanes 1 and 2), together in the dark state (Lane 3) and lit state (Lane 4). The reassembled caspase-7 was reformed after photostimulation (Lane 4). The faint red bands at ~61 kDa and ~56 kDa in Lanes 2, 3, and 4 under the red fluorescence filter is the result of fluorescence bleed-through from the Venus. Lane 1: Casp7C-InN-mRFP; Lane 2: LOVInC-Casp7N-Venus; Lane 3: Co-expression of Casp7C-InN-mRFP and LOVInC-Casp7N-Venus before photostimulation; Lane 4: Co-expression of Casp7C-InN-mRFP and LOVInC-Casp7N-Venus after photostimulation. (TIFF) [file pone.0135965.s003.tiff]

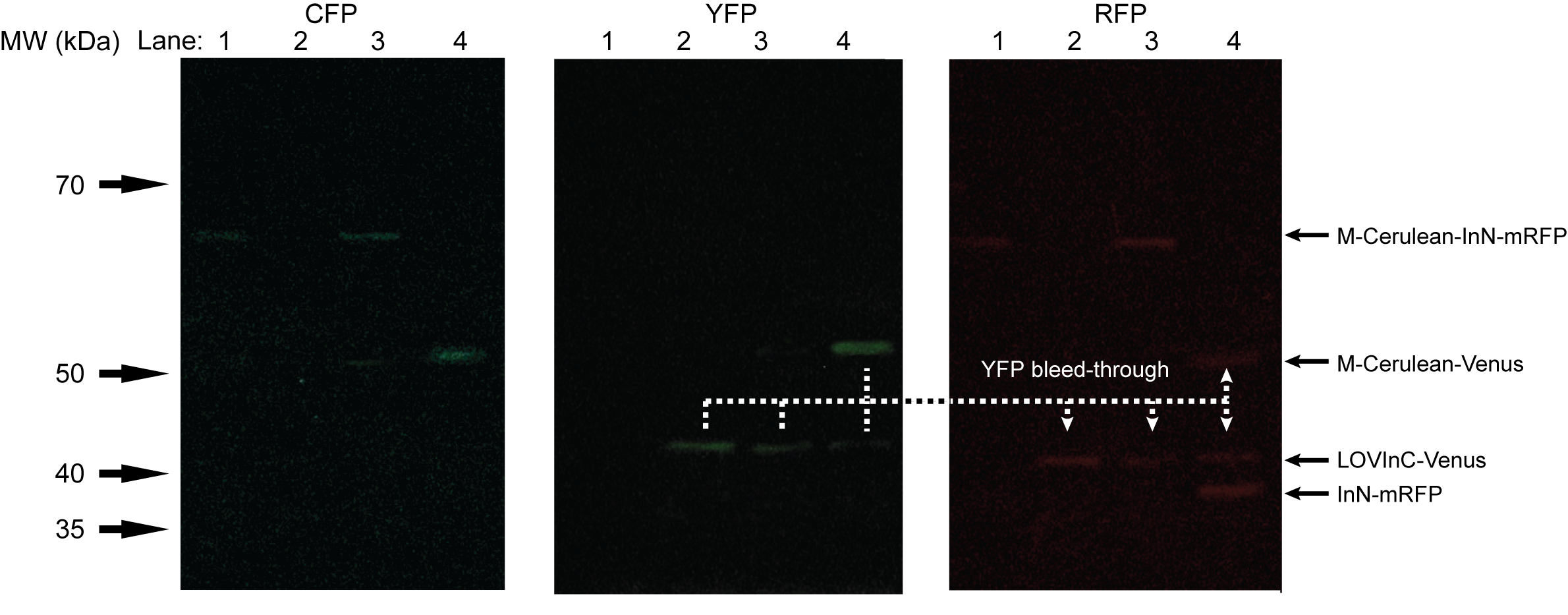

Supplement: S4 Fig — Protein precursors were expressed in E. coli and either grown in the dark (i.e. absence of photostimulation) or grown in the presence of overnight periodic blue-light photostimulation (1 s every 30 s interval). Proteins were extracted by sonication and separated on SDS-PAGE. Fluorescent SDS-PAGE gel separation of constructs expressed separately (Lanes 1 and 2), together in the dark state (Lane 3) and lit state (Lane 4) photo-induced protein trans-splicing. The red band at ~40 kDa in Lanes 1 and 2 under the red fluorescence filter is the result of fluorescence bleed-through from the Venus. Lane 1: M-Cerulean-InN-mRFP; Lane 2: LOVInC-Venus; Lane 3: Co-expression of M-Cerulean-InN-mRFP and LOVInC-Venus before photostimulation; Lane 4: Co-expression of M-Cerulean-InN-mRFP and LOVInC-Venus after photostimulation to yield Lyn-Cerulean-Venus and mRFP-InN. (TIFF) [file pone.0135965.s004.tiff]
